# Transcriptional adaptation of pneumococci and human pharyngeal cells in the presence of a virus infection

**DOI:** 10.1186/1471-2164-14-378

**Published:** 2013-06-07

**Authors:** Sheila Z Kimaro Mlacha, Teresa C T Peret, Nikhil Kumar, Sandra Romero-Steiner, Julie C Dunning Hotopp, Nadeeza Ishmael, Valerie Grinblat-Huse, David R Riley, Dean D Erdman, George M Carlone, Jacquelyn Sampson, J Anthony G Scott, Hervé Tettelin

**Affiliations:** 1 Kenya Medical Research Institute – Wellcome Trust Research Programme, Kilifi, Kenya; 2 Department of Microbiology and Immunology, Institute for Genome Sciences, University of Maryland School of Medicine, Baltimore, MD, USA; 3 Division of Viral Diseases, Centers for Disease Control and Prevention, Atlanta, GA, USA; 4 Division of Bacterial Diseases, Centers for Disease Control and Prevention, Atlanta, GA, USA; 5 Nuffield Department of Clinical Medicine, University of Oxford, Oxford, UK; 6 Present address: Respiratory & Meningeal Pathogens Research Unit, University of the Witwatersrand/Medical Research Council, Johannesburg, South Africa

**Keywords:** *Streptococcus pneumoniae*, RSV, HPIV3, Gene expression, Microarray, Adherence, Bacterial-viral co-infection

## Abstract

**Background:**

Viral upper respiratory tract infections are associated with increased colonization by *Streptococcus pneumoniae* but the mechanisms underlying this relationship are unclear. The objective of this study is to describe a comprehensive picture of the cellular interaction between the adhering bacteria and host cells in the presence or absence of a viral co-infection.

**Results:**

Gene expression profiles of Detroit-562 pharyngeal cells, which were either mock-infected or infected with human respiratory syncytial virus (RSV) or human parainfluenza virus 3 (HPIV3), were analyzed using human microarrays. Transcription response of *S. pneumoniae* strain TIGR4 (serotype 4) in the presence of either mock- or viral-infected cells was analyzed by pneumococcal microarray. Significantly regulated genes were identified by both significance analysis of microarray (SAM) and a ≥ 2-fold change ratio cut-off. The adherence of *S. pneumoniae* to human pharyngeal cells was significantly augmented in the presence of RSV or HPIV3 infection. Global gene expression profiling of the host cells during infection with RSV or HPIV3 revealed increased transcription of carcinoembryonic antigen-related cell adhesion molecules (CEACAM1), CD47, fibronectin, interferon-stimulated genes and many other host cell adhesion molecules. Pneumococci increased transcription of several genes involved in adhesive functions (*psaA*, pilus islet), choline uptake and incorporation (*lic* operon), as well as transport and binding.

**Conclusions:**

We have identified a core transcriptome that represents the basic machinery required for adherence of pneumococci to D562 cells infected or not infected with a virus. These bacterial genes and cell adhesion molecules can potentially be used to control pneumococcal adherence occurring secondary to a viral infection.

## Background

Viruses play a pivotal role in modulating host cells, consequently mediating bacterial superinfection [1-8]. However, the mechanisms responsible for promoting bacterial superinfection are poorly understood. There are two potential explanations: (a) the virus changes the structure of the respiratory tract, paving the way for bacterial pathogens [1,9-12]; or (b) the virus alters the host’s innate immune response, making it more susceptible to bacterial infections [13-17].

Although several studies have demonstrated suppression of the host’s immune response as a potential mechanism for bacterial superinfection, the degree of bacterial infection induced by prior viral infection is indistinguishable from that shown using experimental models without a complete immune system e.g. *in vitro* adhesion assays. This suggests separate or additional mechanisms of action that are independent of immune mechanisms. Studies of polymicrobial interactions have revealed that cellular receptors such as CD14, CD15, CD18, carcinoembryonic antigen-related cell adhesion molecule (CEACAM), macrophage receptor (MARCO), platelet-activating factor (PAFR), fibronectin (FN) and fimbriae-associated receptors are likely to be involved in increased bacterial adherence after viral infection [9,11,14,18-21]. Given the diversity of host receptors, this list of adhesion molecules is unlikely to be exhaustive. Only a few studies have examined the local surface remodeling of human pharyngeal cells by viruses despite the fact that they are the portal of entry for both viruses and bacteria. The full range of adhesion molecules that can be up-regulated during a respiratory viral infection and facilitate bacterial attachment and entry is still unknown.

Some studies suggest that bacterial factors also play a part in this interaction, but whether or not bacteria modulate their surface structures to enhance adherence in the presence of viral infection remains controversial [19,22-24]. Several pneumococcal adhesins have been described [25-27] but their significance in virus-enhanced adherence has not been studied. PspA is the only pneumococcal virulence factor that has been shown to contribute to secondary pneumococcal infections after viral infection [22]. We sought to examine the genes expressed by *Streptococcus pneumoniae* when in contact with virus-infected cells in order to facilitate the design of vaccine and therapeutic targets to control bacterial adherence during polymicrobial infections. We used DNA microarrays to obtain a comprehensive view of: (a) the responses of human pharyngeal cells to infection with respiratory syncytial virus (RSV) and human parainfluenza virus type 3 (HPIV3) and (b) the effect that the viral infection has on both attachment and gene regulation of the pneumococcus.

## Methods

### Bacterial and viral strains and cell lines

TIGR4 [28] and G54 [29] pneumococcal strains, respiratory syncytial virus (RSV) strain ch 93-18b and human parainfluenza virus 3 (HPIV3) strain C243 were used in this study. The RSV strain was originally obtained from the University of Rochester Medical Center and the HPIV3 strain originated from the CDC respiratory repository. Pharyngeal human carcinoma epithelial cells (Detroit 562, CCL138) were obtained from ATCC (Rockville, MD, USA) and were grown and maintained as previously described [26].

### Viral infection scheme and adherence assay

Tissue culture microtiter plates were seeded with 200 μl of a 2 × 10^5^ D562 cells/ml suspension per well and grown for ~4 d to about 80% confluence (9.6 × 10^4^ cells/well). Semi-confluent monolayers were washed twice with phosphate buffered saline (PBS) and inoculated with a 100 μl volume of viral suspension. To infect monolayers with RSV, the virus stock containing 6.5 × 10^6^ TCID_50_/ml was diluted to 10^-1^ to 10^-3^ with minimal essential medium with Eagle's salts (EMEM) (Gibco Laboratories, Grand Island, NY, USA), supplemented with penicillin (50 μg/ml), streptomycin (50 μg/ml) and 2% fetal bovine serum (FBS) (Hyclone, Logan, UT, USA). Each dilution of the virus was inoculated in triplicate. For HPIV3, the virus stock containing 8.3 × 10^7^ TCID_50_/ml was diluted to 10^-3^ to 10^-6^ with serum-free EMEM containing penicillin (100 μg/ml), streptomycin (100 μg/ml) and supplemented with crystallized porcine trypsin. Each virus dilution was inoculated into six wells. The negative control, which constituted of the same culture medium used for viral inoculation but without the virus, was seeded in another six wells. Plates were incubated at 37°C in a 5% CO_2_ incubator and, at 24 h intervals after inoculation (24–72 h for RSV and 24–120 h for HPIV3), were used for pneumococcal adherence experiments as previously described [26]. Briefly, monolayers were washed once with 125 μl/well of EMEM without L-glutamine and supplemented with 7% FBS (Atlas Biologicals, Fort Collins, CO). To the washed monolayer of each well, 80 μl of EMEM was added, followed by 20 μl/well of bacterial suspension (10^3^ bacteria/well). The final D562 cell yield was 1.2 × 10^5^ cells/well after 6 days of incubation resulting in an MOI of 0.01. Plates were incubated for 2 h at 37°C in a 5% CO_2_ incubator to allow for adherence then washed 5 X with PBS with 0.2% bovine serum albumin (BSA) to remove non-adherent pneumococci. A 65 ml volume of Todd-Hewitt broth supplemented with 0.5% yeast extract (THYE), 0.8% agar, and 0.1% 2,3,5-triphenyl tetrazolium chloride (TTC; Difco Laboratories), was added and the plates were incubated overnight at 37°C in a 5% CO_2_ incubator. The number of colonies of *S. pneumoniae* adhering to D562 cells was counted using an automated colony counter (AlphaImager; Alpha Innotech, CA). Adherence to both mock-infected cells (medium with no virus) and virus-infected cells was expressed as the mean (± standard error of the mean, SEM) number of colony forming units (CFUs) in at least 3 replicate wells. Differences in adherence between virus- and mock-infected cells were tested by Student’s *t*-test (*p* < 0.05). RSV or HPIV3 growth was detected and monitored by the use of an indirect immunofluorescence assay (Light Diagnostics Respiratory Viral Screen IFA, Millipore Corporation, Billerica, MA).

It is worth noting that although the adherence assays in this and in the accompanying manuscript by Kimaro Mlacha et al. were performed the same way, there was one difference in the treatment of mock-infected D562 cells (cells not infected with viruses) prior to the adherence assay. For this manuscript, the D562 cells were manipulated before reaching 100% confluence. At day 4, cells were washed to allow for viral inoculation. This manipulation of the monolayer might have resulted in fewer D562 cells being present at day 6 compared than those in the accompanying manuscript, which were left intact for 6 days. Despite this difference, the mock-infected and virus-infected cells within the assay described in this manuscript were treated exactly the same way, making the comparison relevant within this experiment.

For microarray experiments, semi-confluent D562 cells grown in tissue culture flasks were either mock-infected or infected with stock virus at 6.5 × 10^5^ TCID_50_ /ml for RSV and 8.3 × 10^3^ TCID_50_/ml for HPIV3, and incubated at 37°C on a shaker. After 1 h of adsorption, fresh medium was added into each flask and cultures were incubated at 37°C for 3 d. The D562 cells were used for: (a) pneumococcal microarray experiments or (b) analysis of host-cell transcription profiles upon exposure to RSV or HPIV3. (a) Virus-infected and mock-infected cells were inoculated with 1 ml of pneumococcal TIGR4 strain containing 10^7^ bacteria and incubated for 2 h at 37°C in a 5% CO_2_ incubator. Non-adherent bacteria (contained in the spent cell culture medium) were removed and cells were washed 3X with PBS and then treated immediately with 10 ml of RNAprotect (Qiagen, Valencia, CA, USA). Adherent bacteria were dissociated from host cells by lysis with 0.1% (w/v) saponin in PBS followed by sonication using 5 s pulses for 1 min. Bacteria were subsequently harvested by differential centrifugation. Control bacteria, which were not exposed to host cells, were suspended in EMEM medium and then prepared in parallel and treated identically to adherent bacteria. Pellets were stored at −80°C. (b) After inoculation of semi-confluent D562 cells with viruses for 3 d, the viral suspension (or un-inoculated media for mock-infected controls) was removed from the monolayer and the cells were washed twice with PBS with 0.2% BSA. Subsets of the monolayers (both mock and virus-infected) were inoculated with 1 ml of pneumococcal TIGR4 strain suspension containing 10^7^ bacteria, and the remaining monolayers were mock-infected with EMEM. All monolayers were incubated for 2 h at 37°C in a 5% CO_2_ incubator then washed 2X with PBS with 0.2% BSA. D562 cells were detached by treatment with 0.025% trypsin-EDTA (Gibco Laboratories, Grand Island, NY, USA), and cell clumps were disrupted by gently pipetting up and down. The cells were collected by centrifugation at 800 x *g* for 5 min and immediately placed into 5 vol of RNAlater (Ambion, Austin, TX, USA) to minimize RNA degradation. Samples were stored at 4°C overnight to allow RNAlater solution to thoroughly penetrate the cells. The supernatant was then removed and the pellet was stored at −80°C until further processing.

### RNA preparation

Total RNA was isolated from: (a) RNA-stabilized bacteria adherent to either mock- or virus-infected D562 cells and bacteria growing freely in EMEM and (b) monolayers of D562 cells either mock-infected or infected with RSV or HPIV3. RNA was extracted using TRIzol (Invitrogen Life Technologies, Carlsbad, CA, USA) in a lysing matrix containing silica beads on a FastPrep Instrument (MP Biomedicals, Solon, OH, USA) according to the manufacturer’s protocol. RNA was purified with the RNeasy Mini Kit (Qiagen, Valencia, CA, USA) and the quality was assessed using the prokaryote and eukaryote total RNA chips on the Agilent 2100 Bioanalyzer (Agilent Technologies, Santa Clara, CA, USA).

### Microarray experiments

Bacterial microarray experiments were performed on version 6 *S. pneumoniae* DNA microarrays distributed by the Pathogen Functional Genomics Resource Center (PFGRC, J. Craig Venter Institute, Rockville, MD, USA) and consisted of 70-mer oligonucleotides representing open reading frames (ORFs) from the genomes of three strains: TIGR4, G54 and R6, as well as 10 amplicons and 500 70-mer oligonucleotides from *Arabidopsis thaliana*, which served as negative controls. The experiments were performed as previously described [30]. For analysis of the human host cell response, microarrays with PCR amplicons of 41,000 cDNA clones were used (kindly provided by Norman Lee at George Washington University, Washington, DC, USA). Preparation of labeled cDNA target and hybridization experiments were done as previously described [30] with the exception that for the human host cell response, the starting amount of RNA used to synthesize cDNA was 5 μg. Total RNA was isolated from 3 independent cultures (biological replicates) of TIGR4 strain and D562 cells. Dye-swap experiments (technical replicates) were also performed on each biological replicate.

### Data normalization and analysis

Data were analyzed using the TM4 microarray software suite [31]. Spot intensities were quantified using Spotfinder v3.1.1. Normalization was performed using the iterative log mean centering algorithm implemented in the MIDAS software (v2.19), and the fluorescence ratios were calculated from the normalized values. Data from the independent replicate experiments (only where *n* ≥ 15) were averaged using locally developed Perl scripts and the resultant averages used for clustering algorithms to check for similar patterns in gene expression. Hierarchical clustering (HCL) using average linkage and Euclidean distance was also performed on the data to check for variability across replicate slides. To determine whether the observed ratio changes in expression of genes were both statistically and biologically significant, we applied the double filter of: (a) a statistical test, significance analysis of microarrays (SAM) and (b) a threshold ratio of relevant magnitude – a two-fold rise/fall in signal. For SAM analysis, a ∆ value of 1.18 was used for TIGR4 in contact with mock-infected and ∆ = 2.158 for TIGR4 in contact with viral-infected cells. This ∆ cutoff corresponded to a false positive rate of 0%. We also included into the final analysis all statistically significant genes that did not meet the threshold value but appeared to be co-regulated with one of our identified genes as part of an operon. For analysis of the human host cell response to viruses, a less stringent fold change cut-off of 1.5 was used since the overall gene expression changes were generally lower on the human microarrays.

### Validation of human microarray data by qRT-PCR

Reverse transcription was carried out using the QuantiTect Reverse Transcription Kit (Qiagen, Valencia, CA, USA) in accordance with the manufacturer’s instructions. Briefly, 1 μg of total RNA was incubated in gDNA Wipeout Buffer (7X) and RNase-free water and incubated at 42°C for 2 min to remove contaminating genomic DNA. The cDNA was synthesized from the RNA using Quantiscript reverse transcriptase (RT), Quantiscript RT buffer (5X), and a primer mix at 42°C for 15 min and then at 95°C for 3 min to inactivate the Quantiscript RT. Dilutions of the cDNA (0.25 μl of the above mixture per 20 μl reaction) were used as template in a reaction containing 2X QuantiTect SYBR Green mix (Qiagen, Valencia, CA, USA), RNase-free water and gene-specific primers (Additional file 1: Table S1). The qRT-PCR assays were conducted using an ABI 7900HT instrument (Applied Biosystems, Carlsbad, CA, USA). The reactions were denatured at 95°C for 15 min followed by amplification with 45 cycles of 94°C for 15 s, 55°C for 30 s and 72°C for 30 s. Data was analyzed using a comparative cycle threshold (ΔCt) method [32]. The ΔCt was normalized to a gene (NG_007992 - actin gene) that did not exhibit any significant change in expression as identified by the microarray experiments. Each sample was tested in triplicate.

A similar procedure was used for qRT-PCR validation of pneumococcal microarray data. The primers used are listed in Additional file 1: Table S2.

## Results

### Effect of RSV and HPIV3 infection on the adherence of *S. pneumoniae* to human pharyngeal cells

**Figure 1:**
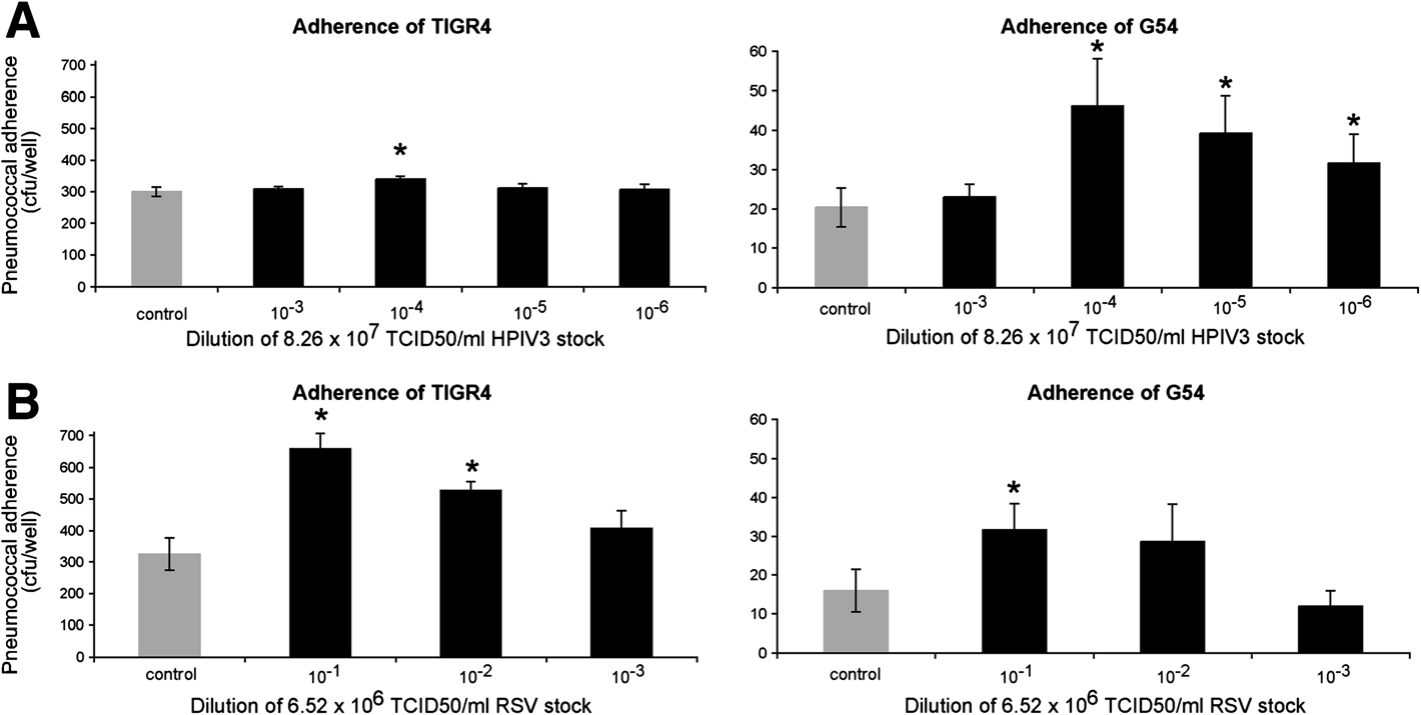
**Binding of *S. pneumoniae *TIGR4 and G54 to D562 cells infected with HPIV3 (A) or RSV (B).** The number of bacteria adhering to mock-infected cells (gray) and to viral-infected cells (black) is shown. The number of bacteria is calculated as the mean (± SEM) number of colony forming units observed in at least 3 replicate wells. Adhesion of pneumococci to viral-infected cells vs. mock-infected at varying viral doses is noted with a star (*p* < 0.05, Student’s test). Since the primary comparison in this figure is between mock-infected and virus-infected cells, the Y-axis scales for the two bacterial strains are different.

D562 cells were permissive to infection with RSV and HPIV3, with discrete cytopathic effect (CPE) after 72 h but without much detachment of the monolayers hence allowing for pneumococcal adherence. We then investigated whether pneumococci adhered in greater numbers to virus-infected D562 cells than to mock-infected cells. The optimal incubation period of virus-infected cells required for maximum bacterial adherence was determined to be 72 h in a preliminary time-course experiment (data not shown). The adherence of pneumococcal strains TIGR4 and G54 to D562 cells increased significantly with prior viral infection (*p* < 0.05) (Figure 1). The degree of enhanced attachment ranged from 1.3- to 2-fold and was dependent on the dose of initial viral infection. Maximal adherence due to RSV infection was obtained at a viral titer of 6.5 × 10^5^ TCID_50_/ml, and maximal adherence to HPIV3 was obtained at a viral titer of 8.3 × 10^3^ TCID_50_/ml. This influence of viral dose on bacterial adherence was similar for both pneumococcal strains, although the strains varied markedly in their baseline adherence capacity without viral infection (Figure 1).

### Gene expression patterns of *S. pneumoniae* in contact with either virus- or mock-infected human pharyngeal cells

To limit the number of variables being compared for the gene expression analysis, we restricted the experiments to one strain of pneumococcus, TIGR4, and one respiratory virus – HPIV3. RNA was isolated from TIGR4 in contact with HPIV3- or mock-infected D562 cells.

Compared to controls (pneumococci in cell culture medium), the gene regulation ratios for pneumococci in contact with virus- or mock-infected pharyngeal cells varied from 0.1 to 14.4. Among the 77 genes that were up-regulated, 51 were regulated in both virus-infected and mock-infected conditions (Additional file 1: Table S3). These included genes involved in adhesive functions (*psaA*, pilus islet), choline uptake and incorporation (*lic* operon), transport and binding (SP_1855-SP_1857) and fatty acid metabolism (SP_0421-SP_0427). The most highly represented genes were those coding for hypothetical proteins (25%), some of which are putative membrane proteins. Of the 98 down-regulated pneumococcal genes, 62 were down-regulated in both virus-infected and mock-infected conditions and these included choline transporters (*proWX, proV*) and purine biosynthesis genes (*pur* operon) (Additional file 1: Table S3). Among these 51 up-regulated and 62 down-regulated genes, the degree of regulation was remarkably consistent in the two adherence conditions (Additional file 1: Figure S1). These genes underpin the common set of cell functions that are induced by exposure to pharyngeal cells – regardless of whether those cells are viral-infected.

Eight pneumococcal genes appeared in the list of genes that were up-regulated after contact with HPIV3-infected cells but were absent in the list of genes up-regulated after contact with mock-infected cells. These were: *glnQ*, a fibronectin-binding/glutamine transport gene, SP_0204-SP_0206, ribonucleotide reductase genes, SP_1428 and SP_2005, genes encoding hypothetical proteins, and SP_1765-SP_1767, glycosyl transferase genes. Upon further examination, we found that these genes were absent from the list of genes up-regulated by TIGR4 in contact with mock-infected cells because they did not meet our criteria for inclusion in the analysis (see Methods). We used qRT-PCR to verify the expression levels of these genes in both conditions, adherence to HPIV3-infected and mock-infected cells. The 8 genes had comparable Ct values between the two conditions. Eighteen genes, all encoding hypothetical proteins, were up-regulated by TIGR4 in contact with mock-infected cells only. It is likely that the expression of these 18 genes is inhibited by the viral infection.

### Effect of HPIV3 and RSV on the expression of human cell adhesion molecules

Given the very large number of human genes and the particular hypothesis under test in this study, we focused our analysis on genes encoding cell adhesion molecules. HPIV3 infection induced the up-regulation of 55 human genes (Table 1A). Four of these were also up-regulated in RSV-infected cells (Table 1 panels A and B, CD47, AADACL1, ZC3HAV1 and cIAP2, an apoptosis inhibitor). Other genes that were up-regulated by HPIV3-infected cells included interferon stimulated genes (ISGs), plasminogen activator urokinase (PLAU), collagen type XVII alpha 1, claudin 1, laminin alpha 3, matrix metallopeptidase 14, and molecules of the carcinoembryonic antigen-related cell adhesion molecules (CEACAM1). Infection with RSV induced the up-regulation of 20 genes, including proteins of the zinc finger family (ZNF23, ZNF318), protocadherin beta 19, Rho family GTPase 1, fibronectin type III domain containing protein, and caspase 10.

**Table 1:** Differential expression of cell adhesion molecules

**A. Genes up-regulated by infection with HPIV3**							
			Fold change				
GB#	Gene name	Gene symbol	HPIV3-infected/mock-infected	RSV-infected/mock-infected	TIGR4-infected/mock-infected	HPIV3-TIGR4-infected/mock-infected	RSV-TIGR4-infected/mock-infected
AA406020	ISG15 ubiquitin-like modifier	ISG15	23.80	1.10	1.05	24.76	1.15
AA456886	Myxovirus (influenza virus) resistance 1, interferon-inducible protein p78	MX1	23.68	1.11	1.04	16.12	1.12
AA286908	Myxovirus (influenza virus) resistance 2	MX2	13.84	1.06	1.05	12.35	1.04
T95113	Radical S-adenosyl methionine domain containing 2	RSAD2	11.72	0.90	1.11	4.49	1.39
AA126958	DEAD (Asp-Glu-Ala-Asp) box polypeptide 58	DDX58	11.55	1.16	0.88	9.94	0.96
AA419251	Interferon induced transmembrane protein 1 (9-27)	IFITM1	7.32	0.96	0.99	5.88	0.76
AA421603	SAM domain and HD domain 1	SAMHD1	5.67	0.96	0.89	4.80	0.86
AI245550	Phospholipid scramblase 1	PLSCR1	5.00	0.91	0.95	3.36	1.11
AA995904	TCF3 (E2A) fusion partner	TFPT	4.05	1.35	1.05	4.12	0.91
AA827287	Interferon-induced protein 35	IFI35	3.85	1.10	0.95	3.40	1.00
H54629	Tumor necrosis factor (ligand) superfamily, member 10	TNFSF10	3.64	NA	1.18	4.32	NA
AA862371	Interferon induced transmembrane protein 2 (1-8D)	IFITM2	3.37	0.85	0.92	2.18	0.93
AA877255	Interferon regulatory factor 7	IRF7	3.25	X	1.06	2.13	0.71
**R07870**	**Baculoviral IAP repeat-containing 3; inhibitor of apoptosis protein**	**-**	**3.09**	**2.09**	**1.20**	**1.79**	**1.60**
AI038270	Eukaryotic translation initiation factor 2-alpha kinase 2	EIF2AK2	2.91	1.11	0.99	1.56	1.04
AA479795	Interferon stimulated exonuclease gene 20kda	ISG20	2.91	NA	1.10	2.29	NA
N67034	Interferon-induced protein 44-like	IFI44L	2.87	1.01	1.05	2.90	0.94
**AA418724**	**Zinc finger CCCH-type, antiviral 1**	**ZC3HAV1**	**2.82**	**1.53**	**1.31**	**1.77**	**1.49**
N75384	Peroxisome proliferator-activated receptor gamma, coactivator 1 beta	PPARGC1B	2.45	0.78	0.94	NA	0.58
W37864	Phosphatase and tensin homolog	PTEN	2.43	0.90	0.93	1.05	0.78
H17861	Ring finger protein 213	RNF213	2.26	1.05	1.03	1.14	1.02
AA983252	Signal transducer and activator of transcription 2, 113kda	STAT2	2.23	X	1.12	1.53	NA
R70479	Tumor necrosis factor, alpha-induced protein 3	TNFAIP3	2.17	0.75	1.03	NA	0.73
AA128561	Collagen, type XVII, alpha 1	COL17A1	1.95	1.11	1.99	1.16	1.15
AI016022	NLR family, CARD domain containing 5	NLRC5	1.93	X	X	X	0.94
N70463	B-cell translocation gene 1, anti-proliferative	BTG1	1.90	1.43	1.13	NA	1.39
H61758	ELK4, ETS-domain protein (SRF accessory protein 1)	ELK4	1.86	0.80	0.87	NA	1.03
R33456	Desmoplakin	DSP	1.82	1.03	0.88	1.18	0.86
**AI085559**	**Arylacetamide deacetylase-like 1**	**AADACL1**	**1.80**	**1.64**	**X**	**1.69**	**1.79**
AA609992	Dehydrogenase/reductase (SDR family) member 9	DHRS9	1.78	1.45	0.95	1.09	1.50
AA136060	Polycomb group ring finger 5	PCGF5	1.74	0.99	0.96	1.21	0.82
AI364513	Scavenger receptor class B, member 2	SCARB2	1.72	1.06	NA	NA	0.97
AA491191	Interferon, gamma-inducible protein 16	IFI16	1.72	1.38	0.94	0.97	1.99
AA451844	Microtubule associated monoxygenase, calponin and LIM domain containing 2	MICAL2	1.70	0.91	0.91	X	0.79
**R92801**	**CD47 molecule**	**CD47**	**1.68**	**1.57**	**1.01**	**1.16**	**1.64**
R93911	Glycogen synthase kinase 3 beta	GSK3B	1.68	1.29	NA	NA	1.32
AA776304	Pleckstrin 2	PLEK2	1.67	0.97	NA	1.81	1.08
AA490894	Endoplasmic reticulum aminopeptidase 1	ERAP1	1.67	1.19	0.87	X	1.25
AA777854	Ring finger protein 12	RNF12	1.67	0.85	0.99	NA	0.92
AA040699	ELK3, ETS-domain protein (SRF accessory protein 2)	ELK3	1.65	0.99	0.91	X	0.86
H95362	Claudin 1	CLDN1	1.64	0.83	NA	1.26	1.20
AA411757	Carcinoembryonic antigen-related cell adhesion molecule 1 (biliary glycoprotein)	CEACAM1	1.63	X	NA	1.41	0.51
AA284668	Plasminogen activator, urokinase	PLAU	1.63	1.15	1.27	X	1.25
AA135422	CCR4-NOT transcription complex, subunit 1	CNOT1	1.63	0.99	0.89	1.04	1.05
AI049712	Epidermal growth factor receptor	EGFR	1.62	1.00	0.91	1.13	0.96
N70848	Ring finger protein 141	RNF141	1.58	1.11	0.94	1.08	0.90
AA005112	LIM domain 7	LMO7	1.58	0.89	0.83	0.81	0.77
AA488674	Myeloid cell leukemia sequence 1 (BCL2-related)	MCL1	1.58	0.96	X	X	0.84
AA018412	Coiled-coil domain containing 93	CCDC93	1.57	0.92	1.11	1.03	0.85
AA478738	Catenin, beta interacting protein 1	CTNNBIP1	1.55	0.97	0.88	X	1.03
AI652954	Transglutaminase 1 (K polypeptide epidermal type I, protein-glutamine-gamma-glutamyltransferase)	TGM1	1.54	0.67	0.86	1.05	0.56
N59721	Serpin peptidase inhibitor, clade E (nexin, plasminogen activator inhibitor type 1), member 2	SERPINE2	1.54	0.69	0.88	X	0.93
AA001432	Laminin, alpha 3	LAMA3	1.53	1.23	0.86	0.94	1.21
AA706099	NEDD4 binding protein 1	N4BP1	1.52	1.08	1.01	1.19	0.98
N33214	Matrix metallopeptidase 14 (membrane-inserted)	MMP14	1.52	0.84	0.79	1.26	0.91
**B. Genes up-regulated by infection with RSV**							
AA406373	Transporter 2, ATP-binding cassette, sub-family B (MDR/TAP)	TAP2	1.46	2.28	1.10	1.18	1.27
AA620877	Protein tyrosine phosphatase, receptor type, M	PTPRM	X	2.11	1.10	1.18	1.27
**R07870**	**Baculoviral IAP repeat-containing 3; inhibitor of apoptosis protein**	**-**	**3.09**	**2.09**	**1.20**	**1.79**	**1.60**
AA701353	Hypothetical protein LOC92270	LOC92270	0.79	2.07	0.90	1.34	2.45
AA001983	Hypothetical LOC92482	LOC92482	0.83	1.74	1.08	NA	X
AI014782	Trinucleotide repeat containing 6B	TNRC6B	1.22	1.99	1.08	0.86	1.88
H80712	Caspase 10, apoptosis-related cysteine peptidase	CASP10	X	1.73	0.79	X	1.84
H23077	Rho family GTPase 1	RND1	X	1.71	X	NA	1.75
AA903644	Protocadherin beta 19 pseudogene	PCDHB19P	0.84	1.65	X	X	1.67
**AI085559**	**Arylacetamide deacetylase-like 1**	**AADACL1**	**1.80**	**1.64**	**NA**	**1.69**	**1.79**
H01197	Pleckstrin homology domain containing, family F (with FYVE domain) member 2	PLEKHF2	0.96	1.63	0.87	0.87	1.35
R36431	Fibronectin type III domain containing 3A	FNDC3A	1.37	1.62	0.92	1.03	1.41
AA047413	Zinc finger protein 23 (KOX 16)	ZNF23	NA	1.60	1.18	X	1.20
AA903552	Lysozyme-like 1, lysozyme-like 2	LYZL1, LYZL2	X	1.58	NA	NA	1.79
AA485438	Ring finger protein 187	RNF187	0.84	1.58	1.02	0.86	1.18
**R92801**	**CD47 molecule**	**CD47**	**1.68**	**1.57**	**1.01**	**1.16**	**1.64**
AI168153	Pleckstrin homology domain containing, family A (phosphoinositide binding specific) member 8	PLEKHA8	X	1.57	X	X	1.41
AA878257	Colony stimulating factor 1	CSF1	X	1.57	NA	NA	1.13
**AA418724**	**Zinc finger CCCH-type, antiviral 1**	**ZC3HAV1**	**2.82**	**1.53**	**1.31**	**1.77**	**1.49**
AI004484	Zinc finger protein 318	ZNF318	X	1.52	1.29	NA	1.62
**C. Genes up-regulated by infection with TIGR4**							
T65736	Selenium-binding protein	SELENBP1	3.98	0.65	1.35	1.12	NA
AI093729	ADAM metallopeptidase with thrombospondin type 1 motif, 2	ADAMTS2	3.87	1.61	1.59	0.59	NA
AA443000	Granulocyte colony stimulating factor receptor	CSF3R	2.75	1.43	0.89	1.10	0.93
AA460304	Human ribosomal DNA complete repeating unit	CRISPLD2	2.58	NA	1.27	NA	0.75
AI003033	Neural cell adhesion molecule 2	NCAM2	2.56	1.50	1.43	1.36	0.97
AA904923	HNF1 homeobox B	HNF1B	2.34	0.65	1.40	NA	1.30
AA521362	CR2/CD21/c3d/Epstein-Barr virus receptor complement component receptor 2	CR2	2.33	NA	1.15	NA	0.99
T98262	General transcription factor IIIC, polypeptide 3	GTF3C3	2.33	0.96	1.50	NA	0.72
AA904604	RIKEN	LOC143678	2.21	1.11	0.80	1.46	0.95
AI188215	Neuregulin 1	NRG1	2.21	NA	0.91	NA	0.80
W60968	Myelin protein zero-like protein2; Epithelial V-like antigen 1 precursor	MPZL2	2.19	NA	2.47	NA	0.36
R88767	Protocadherin 10; protocadherin 20 precursor	PCDH20	2.13	0.72	1.34	NA	0.81
R01281	Src kinase-associated phosphoprotein1; SKAP55 protein	SKAP55	1.94	NA	1.22	NA	1.17
AA150694	LY6/PLAUR domain containing 6	LYPD6	1.85	NA	1.26	NA	1.08
AA707615	Chromosome 9 open reading frame 116	C9orf116	1.83	0.73	1.05	NA	0.87
H57180	Phospholipase C-like phospholipase C, gamma 2 (phosphatidylinositol-specific)	PLCG2	1.82	NA	1.23	NA	1.09
AA883775	Metallaproteinase-disintegrin	ADAM30	1.80	0.32	1.19	NA	2.26
AI361560	Homeo box C9	HOXC9	1.76	0.59	0.91	0.89	0.93
AI380234	C1qr(p) complement component C1q receptor	CD93	1.76	0.55	NA	0.56	NA
R86733	Zinc finger protein	ZNF397	1.74	NA	1.59	NA	1.10
R08109	Alu subfamily J sequence contamination warning entry	ZNF398	1.70	1.02	0.96	0.81	1.05
AA176413	F-box protein Fbx20	FBX20	1.70	0.36	1.08	NA	1.09
H90292	Procollagen type V alpha 2	SERPINA1	1.70	1.77	0.94	1.47	0.76
AA680249	Bactericidal/permeability-increasing protein	BPI	1.69	1.20	2.06	1.24	NA
R43755	Intraflagellar transport protein 57 homolog	ESRRBL1	1.68	1.35	1.28	0.97	1.05
AA620742	Xenotropic and polytropic retrovirus receptor	XPR1	1.67	1.37	0.87	0.82	1.00
AA137073	Integrin, beta-like 1 (with EGF-like repeat domains)	ITGBL1	1.66	0.81	1.03	0.74	0.99
T70368	Integrin, beta 5	ITGB5	1.66	1.03	1.09	1.64	1.01
R76099	Toll-like receptor 3	TLR3	1.62	0.58	0.83	NA	0.85
H52352	Complement factor properdin	CFP	1.62	NA	1.54	NA	0.54
R98903	Scavenger receptor class B, member 1	SCARB1	1.59	NA	1.01	NA	0.73
AA150507	Interleukin 1, beta	IL1B	1.59	1.99	1.81	1.17	0.56
T98612	Alpha-1 type III collagen	COL3A1	1.57	1.74	0.83	NA	1.06
T52330	Interleukin 6 receptor	IL6R	1.57	1.36	1.06	1.07	1.17
AA010600	Nuclear RNA export factor 3	NXF3	1.56	1.58	1.23	0.99	1.00
AA176249	Transforming growth factor, beta 2	TGFB2	1.56	0.64	0.87	NA	1.18
R10099	Stabilin-2	STAB2	1.56	0.98	1.10	1.18	1.21
N32241	Zinc finger protein 160	ZNF160	1.55	NA	1.10	NA	1.06
W94121	Tumor necrosis factor receptor superfamily, member 19	TNFRSF19	1.55	1.17	1.06	0.53	0.92
H93115	Ras association (ralgds/AF-6) and pleckstrin homology domains 1	RAPH1	1.54	1.56	0.72	0.80	NA
AA455067	Non-Ab component of amyloid peptide precursor	SNCA	1.54	NA	1.14	NA	0.93
R68721	Apo-2 ligand, TNF-related apoptosis inducing ligand TRAIL	TNFSF10	1.54	NA	0.83	NA	1.24
AA456622	Wiskott-Aldrich syndrome protein interacting protein	WIPF1	1.54	NA	1.16	NA	1.00
H74265	Protein tyrosine phosphatase, receptor type, C	PTPRC	1.53	NA	1.15	NA	1.20
T68892	Secreted apoptosis related protein 2	SFRP1	1.52	NA	0.95	1.17	1.16
AA780815	Alpha-2 type VIII collagen	COL8A2	1.50	0.98	1.14	1.43	0.83

Genes listed are up-regulated by infection with HPIV3 (A), RSV (B), and/or TIGR4 (C).

X indicates genes that did not meet the criteria established for microarray analysis (see Methods); NA indicates data points that had no single value.

Since the results above suggested that the enhanced expression of various adhesion molecules following HPIV3 and RSV infection may increase the level of adherence of pneumococci, we sought to describe the transcription profiles of cell adhesion molecules exposed to the TIGR4 strain and compared them to the virus-induced transcription profiles. We found that genes that were regulated in the presence of HPIV3 and RSV were not significantly regulated in TIGR4; rather, infection with TIGR4 alone resulted in the up-regulation of a unique set of genes (Table 1C) which included selenium-binding protein 1 (SELENBP1), granulocyte colony stimulating factor receptor (CSF3R), and ADAM metallopeptidases. We also analyzed the transcription of host cells exposed to concurrent stimulation by both viral and bacterial pathogens and found that infection of D562 cells with HPIV3 or RSV followed by TIGR4 induced a similar response to that of infection with HPIV3 or RSV only (Table 1 panels A, B and C).

### Confirmation of microarray results by qRT-PCR

A subset of differentially expressed genes from the human microarray experiments was selected and qRT-PCR analysis was undertaken to confirm the relative levels of gene expression. The experiments were performed on the same RNA samples used in microarray experiments. The correlation coefficient (R) between qRT-PCR and microarray results for pneumococcal transcription in contact with either virus-infected or mock-infected epithelial cells was 0.92 and 0.84, respectively. On the host side, the correlation coefficient between the two conditions in HPIV-3 infected cells and RSV-infected cells were 0.87 and 0.65, respectively (Additional file 1: Figure S2).

## Discussion

### Binding of pneumococci to virus-infected cells

In the present study, we have used a well-established model of adherence to demonstrate that infection of human pharyngeal cells with paramyxoviruses RSV or HPIV3 enhanced the adherence of *S. pneumoniae* strains TIGR4 and G54. While this finding is consistent with the results of other *in vitro* studies [21,33-35], this is the first report of RSV and HPIV3 increasing the *in vitro* adherence of *S. pneumoniae* to human pharyngeal cells (Detroit 562). The enhanced adherence of bacteria to virus-infected cells was consistent across both viruses and pneumococcal strains/serotypes evaluated. HPIV3 and RSV were used in this study as they are among the viruses most commonly predisposing to secondary bacterial infections [36,37]. The results also show that both the neuraminidase (NA)-positive (HPIV3) and NA-negative (RSV) viruses enhanced binding of pneumococci to pharyngeal cells. Viral neuraminidase cleaves terminal sialic acid on eukaryotic cells, unmasking receptors that facilitate pneumococcal adherence [38]. This suggests that NA-enhanced bacterial adherence is not the only mechanism for increased bacterial adherence following viral infection.

### Mechanism of viral/bacterial synergy: a bacterial standpoint

We wanted to determine whether: (i) the pneumococcus is capable of detecting and responding to the host cell changes by expressing additional factors to complement newly expressed host cell receptors on the surface of viral-infected cells, or (ii) *S. pneumoniae* simply expresses the same baseline set of genes in response to contact with host cell regardless of the status of viral infection. In the latter case, the advantage of viral infection to pneumococcal adherence would be determined entirely by the viral effect on human cells. Our results demonstrate that there is a common set of pneumococcal genes that participate in the induction of adherence regardless of the presence of a virus. We believe that this study is the first to analyze the reciprocal response of *S. pneumoniae* to viral infection at a global transcriptome level.

As enhanced adherence was observed with both pneumococcal strains and with both viruses, we focused the pneumococcal microarray experiments on one strain–virus combination. We selected TIGR4 because it exhibited greater adherence to epithelial cells than G54, and we selected HPIV3 because it expresses NA. Our observation that multiple pneumococcal adhesins are regulated upon the binding of the bacterium to both mock- and virus-infected cells suggests that there is a common set of genes whose protein products represent good targets for designing interventions to prevent pneumococcal infection in the nasopharynx.

The up-regulated operon SP_0204–SP_0206 encodes a ribonucleoside triphosphate reductase (*nrdD*) and its activating protein (*nrdG*). This operon is active during anaerobic conditions [39,40] and in many bacterial species, the operon is up-regulated to enable bacteria to survive during oxidative stress [39]. The gene *nrdG* has been shown to be up-regulated in *S. pneumoniae* during infection of blood and meninges [41]. Human cells infected with viruses generate a large amount of inflammatory products and therefore up-regulation of this operon in the pneumococcus suggests the existence of oxygen tension. This is a plausible explanation of how the pneumococcus is able to resist this stressful environment and in the end confer the phenotype of enhanced adherence. McCullers and colleagues have proposed that fibrin and fibrinogen, deposited during the regenerative process following viral infection, may provide additional attachment sites for bacteria [2]. It was therefore interesting to observe that *glnQ* (SP_1242), a gene previously shown to be required for adherence to fibronectin by group B *Streptococcus*[42] was significantly up-regulated in TIGR4 in contact with virus-infected cells. It is possible that it might have a specific adhesive function in *S. pneumoniae* on contact with virus-infected epithelial cells.

### Mechanism of viral/bacterial synergy: a host standpoint

We hypothesized that viral infection can enhance bacterial adhesion by increasing the expression of host cell receptors. Our study supported this contention by showing that a variety of epithelial cell receptors (CEACAM1, CD47, interferon-stimulated genes, among others) were up-regulated in response to HPIV3 and RSV infection. Other studies examining this question have either focused exclusively on the expression of a small number of cell adhesion receptors, or have studied non-human epithelial cells. CEACAM1 and CD47 have previously been identified in the host cell response to viral infection [21] and we have confirmed this observation with regard to HPIV3. CEACAM1 binds meningococcal and gonococcal opacity-associated (Opa) proteins and mediates internalization of the bacteria into several cell types *in vitro*[43]. It also binds to *Haemophilus influenzae* and *Moraxella catarrhalis*, which share their ecological niche with *S. pneumoniae*. Though the CEACAM-binding adhesins in *S. pneumoniae* are unknown, if they exist, the fact that CEACAM1 is one of a small number of genes up-regulated after viral infection suggests that this may be an area for future study. CD47, a gene encoding an integrin-associated protein, was up-regulated in response to infection by both HPIV3 and RSV in our study. Expression of CD47 has previously been shown to render mice susceptible to *E. coli* K1 meningitis [44].

Interestingly, the gene encoding a fibronectin type III domain-containing protein was up-regulated by the host in response to both viruses (significantly in RSV and moderately in HPIV3). In our pneumococcal gene expression studies, a fibronectin-binding gene, *glnQ* was highly up-regulated in TIGR4 in contact with virus-infected cells. The symmetry of these observations suggests that virus-induced up-regulation of fibronectin type III induced a reciprocal response in *S. pneumoniae* in the form of up-regulation of a ligand to match the newly up-regulated host receptor. This is a specific hypothesis worth pursuing in future functional experiments.

Other genes that were significantly up-regulated in our study include several interferon-stimulated genes (ISGs) and the urokinase plasminogen activator (PLAU), which mediates a response to the inflammation induced by the virus. Induction of ISGs and NOD-like receptor family (Table 1) in our study is consistent with a recent finding that the cross-talk between Nod1/Nod2 receptors and type 1 IFNs induced during a viral infection, promoted lethality in mice superinfected with *E. coli*[45]. Up-regulation of genes encoding a matrix metalloproteinase, MMP14 and also caspase 10 may provide a mechanism by which the viruses induce lysis of the host cell components and, in the process, facilitate increased adherence and possibly invasion of bacteria.

## Conclusions

In summary, we have shown that: (a) D562 cells were permissive to RSV and HPIV3; (b) these viruses augmented the adherence of *S. pneumoniae* to the host cells by a factor of 1.3-2.0; (c) infection with both RSV and HPIV3 enhanced the transcription of host cell adhesion molecules that facilitate or mediate bacterial adhesion in general; and (d) there is a core transcriptome (at least in the TIGR4 strain) that represents the basic machinery required for adherence of pneumococci to D562 cells regardless of whether the cell is infected with a virus or not. Further elucidation of these mechanisms is most likely to be obtained by specific inhibition of the expression of the host cell receptors or by bacterial gene knockout experiments.

## Abbreviations

CEACAM: Carcinoembryonic antigen-related cell adhesion molecule; CPE: Cytopathic effect; D562 cells: Detroit 562 cells; FN: Fibronectin; HCL: Hierarchical clustering; HPIV3: Human parainfluenza virus 3; ISGs: Interferon-stimulated genes; MARCO: Macrophage receptor; NA: Neuraminidase; PAFR: Platelet-activating factor; RSV: Respiratory syncytial virus; SAM: Significance analysis of microarrays.

## Competing interests

JAGS reports receiving a grant from GlaxoSmithKline Biologicals (Anthony Scott, Kayla Laserson; $2,575,975; Oct 2010-Sep 2013) for a study entitled: ‘A phase IV multi-site observational epidemiology study to assess potential risk for adverse events following immunization that may be associated with misuse of a two-dose vial of 10-valent Pneumococcal Conjugate Vaccine (Synflorix) in Kenya’.

## Authors’ contributions

SZKM carried out the adherence assays and pneumococcal microarray experiments, analyzed the data and drafted the manuscript. TCTP participated in the design and performance of viral adherence assays. NK performed the human microarray experiments and qRT-PCR experiments. SRS participated in the design and performance of bacterial adherence assays and RNA extractions and drafting of the manuscript. JCDH participated in the design and analysis of all experiments involving molecular work in this manuscript. NI participated in the pneumococcal microarray experiments. VGH performed qRT-PCR experiments. DRR participated in the bioinformatics analysis of microarray work. DDE, GMC, JS participated in the design of the study. JAGS and HT conceived the study, participated in the study design and development, analysis and drafting of the manuscript. All authors read and approved this manuscript.

## Supplementary Material

Additional file 1 **Table S1. **is a table listing the primers used for qRT-PCR analysis of human genes. **Table S2.** is a table listing the primers used for qRT-PCR analysis of pneumococcal genes. **Table S3.** is a table listing genes commonly significantly regulated by TIGR4 in contact with either viral- or mock-infected human pharyngeal cells. **Figure S1.** is a figure depicting the correlation of the log_2_ ratios of TIGR4 adherent to mock-infected cells and to HIPV3-infected cells. **Figure S2.** is a figure showing graphs of qRT-PCR validation of human and pneumococcal microarray results. Click here for file
