# Supplementary material for: Transcriptional adaptation of pneumococci and human pharyngeal cells in the presence of a virus infection
Source: BMC Genomics. 2013 Jun 7;14:378. doi: 10.1186/1471-2164-14-378 (PMC3681581; doi:10.1186/1471-2164-14-378)

Table S1 Primers used for qRT-PCR analysis of human genes.

Locus	F-Primer	R-Primer	Prod Size
T64956	ATATTCAGCCTTGCCCTGAG	GCTTAAGAGTGGCTTTCTAGGC	146
R72244	TTTAAGGCTAAAGCCCAAGG	CAAGCTCTCCAAAGGCTCAG	146
AA406020	TCCTGGTGAGGAATAACAAGGG	GTCAGCCAGAACAGGTCGTC	124
T72877	AAACAGAGCTGAAGTCACAGGA	TGTAGAGCTTCTGGCACTTGGA	198
AA432030	TAGGCAGGAGTCCAGAGGAG	ACAGCTCCTGGAACACAACC	153
AI066560	AAGGGCGGGCTATACTAAGG	GTGGAGGTGATGATCAGTGC	100
AA401724	TGGGATCCCTACTCCTCTTG	AAGGAAGGAGACCAGGAAGC	175
W86214	TCAGGTGACACTTCGGTGAC	TTGTGGGTAGATCCCTCTCG	125
AI244834	CTGGTCTTGCTTCAGTGTGC	GAAAGAGGCGGACAGATGAG	109
H93482	GGAAAAATCTGATTCCAGCATTC	AGAGCCTGGACCCATGTAAG	191
AA973391	AGGGCTGATTGCAAATGAAG	CAGGTGCCAGAGCAAGAGTC	137
AA931124	CGACTCCTCTAAGGGCAAAC	TGGATGCATTGTACCATAGGC	121
AI246160	CGTAGACATACCTACCCATTCTTG	CAGCTCAGATCCCGCATATC	126
AA679571	AACTGGCTGGAATGTGCTG	ATCAGTTGCCACTCGGATG	144
AA664101	GGACAATGCTGTTGAATTTGC	CAAACTCATCATAAATTGATTCTTCC	107
AA402898	TTTGAGCGTCGCAGAGAACTT	TGTCCCAAGGATTCTTCCTCC	173
AA034945	AAGGCAAGAACAAATGATTCAC	TTTCAGATATCCAAAGCTTACCAC	103
H92621	ACCTGTCAAGTACCCAAGACT	CGAGGACCACTTAACCTCCAG	108
AA970731	CTTCCCTGCTCCAAGTTCTG	ACTGCTGCATCTTGGGTCTC	115
AA436384	GTGTCTACTCCAGGCCAAGC	GAGCCAGAGCTTGGTGTCTC	131
AA969184	AGTAGAGGAACTGGTCACTGG	TGGGCTAGAAGTGTCTTCAGAT	138
AA442095	AAATTTGAGTTCTGATGAAATGC	ACTGCGGGACAGTCAGTAGG	149
R55786	CATTTAATCATTTGCTGTTCCAAG	CCAAATTGTTGCATCTGGTTC	171
AA447746	GGGATCACTTGGGATCTTTG	AGACCCAGATGGCACTCCTC	153
AA425379	CACTGGCATTTACAATCCTTTC	CCTGGAGACCCTGTCACATC	150
AI340216	AATTATGGCTGGGTGTGGTG	TGGTGTGATCATGGCTTATTTC	114
AI128885	CATCTGCTTCCAACCTCTCC	GGACAAGCAGGCGAATTAAG	168
AI188107	TACTTCCTCACCGGGATGTC	TTTCACCACATGGCACAATC	190
NG_007992	CACTCATTCAACAGCGAGGA	GTTTTAGCCTCTGCAGCCAC	166

Table S2 Primers used for qRT-PCR analysis of pneumococcal genes.

Locus	F-Primer	R-Primer	Prod Size
SP_0204	GGCTACTCCATTCGTCCATC	ATAACGCTCGACTCCATTGC	195
SP_0205	TGGCATTCCCTATACAGCAG	CTTGTCTGGCAATTCCTTCC	157
SP_0206	TGGGAATATACGGTCATGGAG	GCAAACTCTCCAGTTCATGC	198
SP_1242	ATTGGGAATGGAGTTGCTTG	TCAAGGGCAGAAGTTGGTTC	159
SP_1428	TGATAGAGGCACAGGGTTTC	TGAATCATGACACCGATAACAAG	131
SP_1765	TTTGGATTGGTCAGAGATTGC	AGAACAATCACACGGTGCAG	178
SP_1767	GAAGGTGCGACTTCTCGTTC	CAAGCGACCACCAGCATAC	147
SP_2005	CCTTTCAACCACTCCATTCTC	TTATCGGCCTGTCACTCTTG	148
**Controls for normalization**			
SP_2135	AACACAAAGAATCTGGTGAACG	AACACAACGTGTTTGCGAAG	112
SP_0002	TTCCTAGCCGTTCTCTACGC	TGTTGGAATCAAGCGATCTG	167

Table S3 Genes commonly significantly regulated by TIGR4 in contact with either viral- or mock-infected human nasopharyngeal cells. The ratio index is the ratio between "TIGR4 viral infected/TIGR4 in culture medium" and "TIGR4 mock-infected/TIGR4 in culture medium". A ratio index of 1 indicates that the gene expression ratio was the same in both conditions.

Up-regulated genes					
			Fold change		
Locus	Common name	Gene symbol	TIGR4 mock-infected/ TIGR4 in culture medium	TIGR4 viral infected/ TIGR4 in culture medium	Ratio Index
SP_0099	Hypothetical protein		2.2	1.7	0.77
SP_0100	Hypothetical protein		2.2	1.6	0.73
SP_0102	Glycosyl transferase		1.9	2.2	1.16
SP_0103	Putative capsular polysaccharide biosynthesis protein		1.6	1.5	0.94
SP_0371	Hypothetical protein		2	2.1	1.05
SP_0421	3-oxoacyl-[acyl-carrier protein] reductase		2.7	1.5	0.56
SP_0422	3-oxoacyl-(acyl-carrier-protein) synthase II	*fabF*	2.4	1.9	0.79
SP_0423	Hypothetical protein		3.6	1.9	0.53
SP_0424	Putative hydroxymyristoyl-(acyl carrier protein) dehydratase		3.6	2	0.56
SP_0425	Acetyl-coa carboxylase, biotin carboxylase		4.2	2.3	0.55
SP_0426	Acetyl-coa carboxylase, carboxyl transferase subunit beta	*accD*	5.5	3	0.55
SP_0427	Acetyl-coa carboxylase, carboxyl transferase subunit alpha	*accA*	2.5	1.6	0.64
SP_0461	Transcriptional regulator, putative		2.1	2.7	1.29
SP_0462	Cell wall surface anchor family protein		2.6	2.2	0.85
SP_0463	Cell wall surface anchor family protein		1.9	2.2	1.16
SP_0464	Cell wall surface anchor family protein		2.1	2	0.95
SP_0466	Sortase, putative		2.0	2.5	1.25
SP_0467	Sortase, putative		2.3	2.3	1.00
SP_0468	Sortase, putative		2.2	2.3	1.05

Up-regulated genes					
			Fold change		
Locus	Common name	Gene symbol	TIGR4 mock-infected/ TIGR4 in culture medium	TIGR4 viral infected/ TIGR4 in culture medium	Ratio Index
SP_0696	Hypothetical protein		2.1	1.4	0.67
SP_0697	ABC transporter, ATP-binding protein, authentic point mutation		1.8	1.5	0.83
SP_0879	Hypothetical protein		2	2.1	1.05
SP_1261	Hypothetical protein		1.5	1.4	0.93
SP_1267	LicC protein	*licC*	4	1.7	0.43
SP_1268	LicB protein	*licB*	4	1.9	0.48
SP_1269	Choline kinase		3.1	2	0.65
SP_1270	Hypothetical protein		3.1	2.1	0.68
SP_1272	Repeating unit transporter		1.6	1.4	0.88
SP_1273	LicD1	*licD1*	1.3	1.4	1.08
SP_1274	LicD2	*licD2*	1.4	1.3	0.93
SP_1294	Hypothetical protein		2	1.6	0.80
SP_1295	Hypothetical protein	*crcB*	1.8	1.5	0.83
SP_1296	Purtative chorismate mutase protein		1.9	1.6	0.84
SP_1429	YrrO protein	yrrO	2.1	2	0.95
SP_1648	Manganese ABC transporter, ATP-binding protein	*psaB*	2.8	2.2	0.79
SP_1649	Manganese ABC transporter, permease protein, putative, authentic frameshift	*psaC*	2.4	1.3	0.54
SP_1650	Manganese ABC transporter, manganese-binding adhesion liprotein	*psaA*	2	1.6	0.80
SP_1758	Glycosyl transferase, group 1		2.2	1.6	0.73
SP_1770	Glycosyl transferase, family 8		2.2	1.8	0.82
SP_1771	Glycosyl transferase, family 2-glycosyl transferase family 8		1.8	1.7	0.94
SP_1811	Tryptophan synthase, alpha subunit		1.9	2	1.05
SP_1813	N-(5'-phosphoribosyl)-anthranilate isomerase		2	1.8	0.90

Up-regulated genes

Locus	Common name	Gene symbol	Fold change: TIGR4 mock-infected/ TIGR4 in culture medium	Fold change: TIGR4 viral infected/ TIGR4 in culture medium	Ratio Index
SP_1814	Indole-3-glycerol phosphate synthase		1.8	1.9	1.06
SP_1855	Dehydrogenase		6	5.4	0.90
SP_1856	Transcriptional regulator, MerR family		7.6	6.8	0.89
SP_1857	Cation efflux system protein		14.4	13.5	0.94
SP_1922	Conserved hypothetical protein		2.3	1.6	0.70
SP_1923	Pneumolysin	*ply*	2.9	1.9	0.66
SP_1924	Hypothetical protein		2.6	1.7	0.65
SP_1925	Hypothetical protein		2.3	1.5	0.65
SP_2236	Putative sensor histidine kinase ComD	*comD*	2.3	2	0.87

Down-regulated genes

Locus	Common name	Gene symbol	Fold change: TIGR4 mock-infected/ TIGR4 in culture medium	Fold change: TIGR4 viral infected/ TIGR4 in culture medium	Ratio index
SP_0045	Putative phosphoribosylformylglycinamidine synthase		0.2	0.2	1.00
SP_0046	Amidophosphoribosyltransferase		0.4	0.4	1.00
SP_0047	Phosphoribosylformylglycinamide cyclo-ligase		0.4	0.6	1.50
SP_0048	Phosphoribosylglycinamide formyltransferase homolog (fragment)		0.5	0.6	1.20
SP_0049	VanZ protein, putative	vanZ	0.6	0.6	1.00
SP_0050	Phosphoribosylaminoimidazolecarboxamide formyltransferase-imp	*purH*	0.5	0.5	1.00

Down-regulated genes					
			Fold change		
Locus	Common name	Gene symbol	TIGR4 mock-infected/ TIGR4 in culture medium	TIGR4 viral infected/ TIGR4 in culture medium	Ratio index
	cyclohydrolase				
SP_0051	Phosphoribosylamine glycine ligase		0.3	0.3	1.00
SP_0053	Phosphoribosylaminoimidazole carboxylase, catalytic subunit	*purE*	0.4	0.4	1.00
SP_0054	PurK protein (fragment).	purK	0.4	0.5	1.25
SP_0083	DNA-binding response regulator		0.5	0.5	1.00
SP_0084	Sensor histidine kinase		0.4	0.4	1.00
SP_0095	Conserved hypothetical protein		0.2	0.4	2.00
SP_0267	N5,n10-methylenetetrahydromethanopterin reductase, putative		0.3	0.4	1.33
SP_0285	Alcohol dehydrogenase, zinc-containing		0.4	0.3	0.75
SP_0287	Xanthine-uracil permease family protein		0.2	0.3	1.50
SP_0288	Conserved hypothetical protein		0.4	0.4	1.00
SP_0289	Dihydropteroate synthase		0.5	0.7	1.40
SP_0290	Dihydrofolate synthetase		0.7	0.7	1.00
SP_0291	Gtp cyclohydrolase I		0.7	0.8	1.14
SP_0292	Bifunctional folate synthesis protein		0.6	0.8	1.33
SP_0330	RegR	regR	0.5	0.4	0.80
SP_0726	ThiD	thiD	0.3	0.3	1.00
SP_0730	Pyruvate oxidase	*spxB*	0.3	0.4	1.33
SP_0766	Manganese superoxide dismutase.		0.3	0.4	1.33
SP_0784	Glutathione reductase		0.1	0.1	1.00
SP_0820	ATP-dependent Clp protease, ATP-binding subunit ClpE	*clpE*	0.4	0.4	1.00
SP_0867	ABC transporter, ATP-binding protein		0.3	0.4	1.33

Down-regulated genes

Locus	Common name	Gene symbol	Fold change: TIGR4 mock-infected/ TIGR4 in culture medium	Fold change: TIGR4 viral infected/ TIGR4 in culture medium	Ratio index
SP_0868	Conserved hypothetical protein		0.3	0.3	1.00
SP_0869	YurW protein	yurW	0.3	0.3	1.00
SP_0870	Hypothetical protein		0.3	0.4	1.33
SP_0871	YurU protein	yurU	0.3	0.3	1.00
SP_0999	Cytochrome c-type biogenesis protein CcdA	*ccdA*	0.3	0.2	0.67
SP_1000	Hypothetical protein		0.2	0.2	1.00
SP_1045	Conserved hypothetical protein		0.4	0.5	1.25
SP_1227	Response regulator		0.5	0.5	1.00
SP_1228	YfhQ protein	yfhQ	0.3	0.4	1.33
SP_1229	Formate etrahydrofolate ligase		0.3	0.4	1.33
SP_1499	Bacterocin transport accessory protein	*bta*	0.2	0.2	1.00
SP_1546	Hypothetical protein		0.1	0.1	1.00
SP_1587	Oxalate-formate antiporter		0.2	0.2	1.00
SP_1588	Oxidoreductase, pyridine nucleotide-disulfide, class I		0.1	0.1	1.00
SP_1651	Thiol peroxidase	*psaD*	0.3	0.3	1.00
SP_1774	Transcriptional regulator, putative		0.5	0.5	1.00
SP_1775	Hypothetical protein		0.2	0.2	1.00
SP_1776	Thioredoxin reductase	*trxA*	0.2	0.2	1.00
SP_1778	Water channel protein		0.5	0.4	0.80
SP_1845	Exodeoxyribonuclease	*exoA*	0.4	0.4	1.00
SP_1846	Conserved hypothetical protein, degenerate		0.4	0.4	1.00
SP_1848	Xanthine permease		0.3	0.4	1.33

Down-regulated genes					
			Fold change		
Locus	Common name	Gene symbol	TIGR4 mock-infected/ TIGR4 in culture medium	TIGR4 viral infected/ TIGR4 in culture medium	Ratio index
SP_1860	Choline transporter	*proWX*	0.2	0.2	1.00
SP_1861	Choline transporter	*proV*	0.2	0.2	1.00
SP_1862	Hypothetical protein		0.2	0.2	1.00
SP_1907	Cochaperonin groES.		0.4	0.5	1.25
SP_1946	Putative transcriptional regulator PlcR	*plcR*	0.4	0.5	1.25
SP_2054	Hypothetical protein		0.3	0.3	1.00
SP_2055	Alcohol dehydrogenase, zinc-containing		0.3	0.3	1.00
SP_2136	PcpA	*pcpA*	0.5	0.4	0.80
SP_2187	Hypothetical protein		0.3	0.4	1.33
SP_2188	Hypothetical protein		0.5	0.5	1.00
SP_2197	ABC transporter, substrate-binding protein, putative		0.4	0.5	1.25
SP_2198	ABC transporter, permease protein		0.5	0.4	0.80
SP_2199	Hypothetical protein		0.4	0.5	1.25

Figure S1 Correlation of the Log_2 ratios of TIGR4 adherent to mock-infected cells (y-axis) and TIGR4 adherent to HPIV3-infected cells (x-axis) of the commonly regulated genes. The axes are the log_2 ratios for pneumococcal transcription when exposed to HPIV3-infected cells (x-axis) or uninfected cells (y-axis).

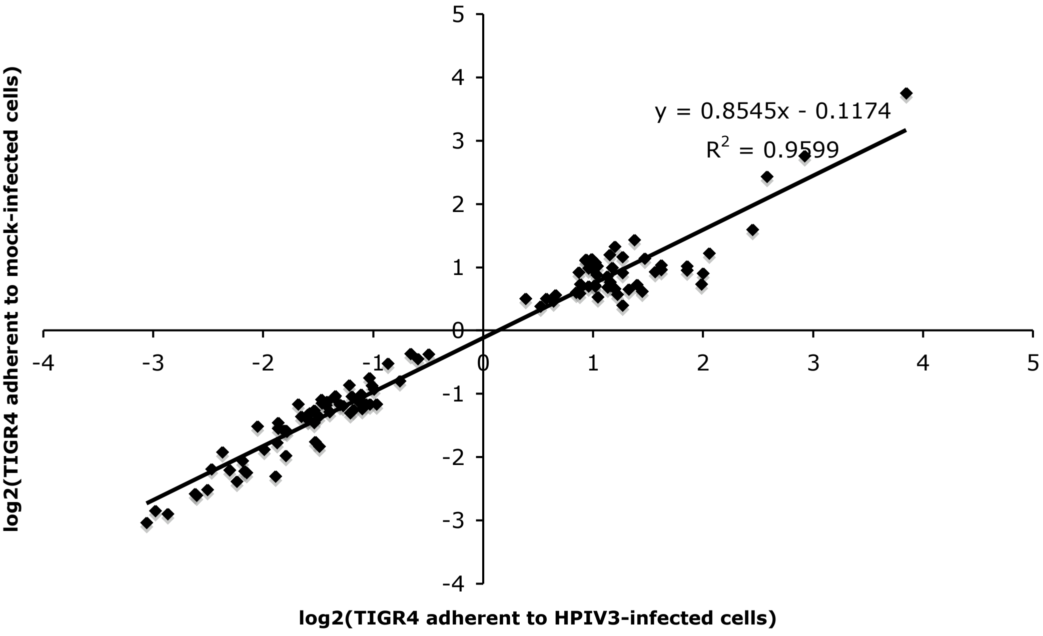

Figure S2 qRT-PCR validation of microarray results. All graphs display log_2 of microarray data on the X axis and log_2 of qRT-PCR data on the Y axis. A. TIGR4 genes regulated upon adherence to mock-infected D562 cells (R=0.84); B. TIGR4 genes regulated upon adherence to HPIV3-infected D562 cells (R=0.92); C. Human genes regulated upon infection of D562 cells with HPIV3 (R=0.87); D. Human genes regulated upon infection of D562 cells with RSV (R=0.65).

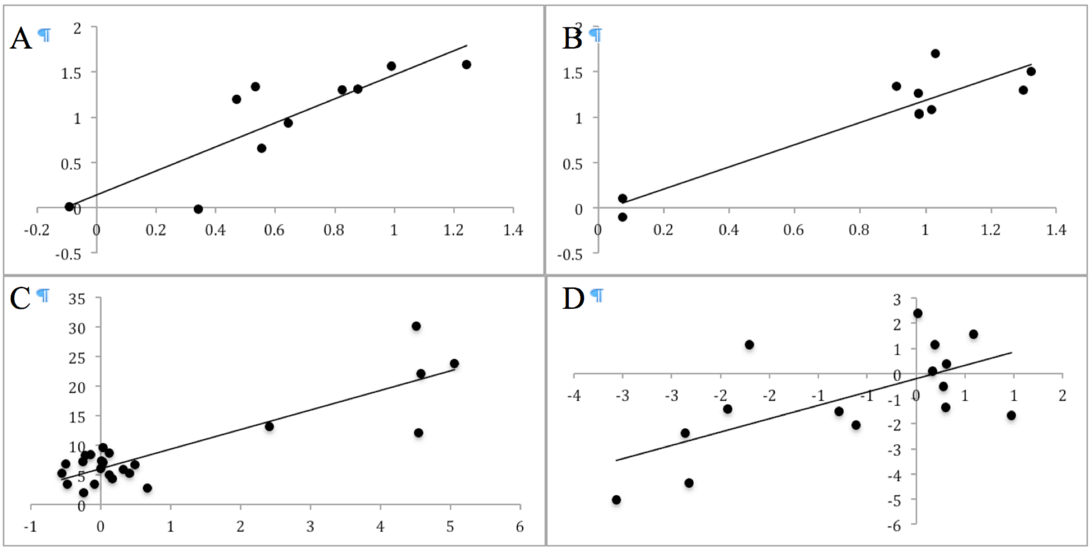

Supplement: Additional file 1 — Table S1. is a table listing the primers used for qRT-PCR analysis of human genes. Table S2. is a table listing the primers used for qRT-PCR analysis of pneumococcal genes. Table S3. is a table listing genes commonly significantly regulated by TIGR4 in contact with either viral- or mock-infected human pharyngeal cells. Figure S1. is a figure depicting the correlation of the log2 ratios of TIGR4 adherent to mock-infected cells and to HIPV3-infected cells. Figure S2. is a figure showing graphs of qRT-PCR validation of human and pneumococcal microarray results. [file 1471-2164-14-378-S1.pdf]
